# Modulation of cytokine and nitric oxide by mesenchymal stem cell transfer in lung injury/fibrosis

**DOI:** 10.1186/1465-9921-11-16

**Published:** 2010-02-08

**Authors:** Shin-Hwa Lee, An-Soo Jang, Young-Eun Kim, Ji-Yeon Cha, Tae-Hoon Kim, Seok Jung, Seong-Kyu Park, You-Kyoung Lee, Jong-Ho Won, Yong-Hoon Kim, Choon-Sik Park

**Affiliations:** 1 Genome Research Center for Allergy and Respiratory Diseases, Division of Allergy and Respiratory Medicine, Soonchunhyang University Bucheon Hospital, 1174, Jung Dong, Wonmi Ku, Bucheon, Gyeonggi Do, 420-767, Korea; 2 Division of Hematology, Soonchunhyang University Bucheon Hospital, 1174, Jung Dong, Wonmi Ku, Bucheon, Gyeonggi Do, 420-767, Korea; 3 Clinical Laboratory Medicine, Soonchunhyang University Bucheon Hospital, 1174, Jung Dong, Wonmi Ku, Bucheon, Gyeonggi Do, 420-767, Korea; 4 Division of Hematology, Soonchunhyang University Seoul Hospital, 657-58, Hannam-dong, Yongsan-gu, Seoul, 140-743, Korea; 5 Division of Allergy and Respiratory Diseases, Soonchunhyang University Hospital, 23-20, Bongmyung-dong, Cheonan city, Choongnam, 330-721, Korea

## Abstract

**Background:**

No effective treatment for acute lung injury and fibrosis currently exists. Aim of this study was to investigate the time-dependent effect of bone marrow-derived mesenchymal stem cells (BMDMSCs) on bleomycin (BLM)-induced acute lung injury and fibrosis and nitric oxide metabolites and inflammatory cytokine production.

**Methods:**

BMDMSCs were transferred 4 days after BLM inhalation. Wet/dry ratio, bronchoalveolar lavage cell profiles, histologic changes and deposition of collagen were analyzed.

**Results:**

Nitrite, nitrate and cytokines were measured weekly through day 28. At day 7, the wet/dry ratio, neutrophilic inflammation, and amount of collagen were elevated in BLM-treated rats compared to sham rats (*p *= 0.05-0.002). Levels nitrite, nitrate, IL-1β, IL-6, TNF-α, TGF-β and VEGF were also higher at day 7 (*p *< 0.05). Degree of lymphocyte and macrophage infiltration increased steadily over time. BMDMSC transfer significantly reduced the BLM-induced increase in wet/dry ratio, degree of neutrophilic infiltration, collagen deposition, and levels of the cytokines, nitrite, and nitrate to those in sham-treated rats (*p *< 0.05). Fluorescence *in situ *hybridization localized the engrafted cells to areas of lung injury.

**Conclusion:**

Systemic transfer of BMDMSCs effectively reduced the BLM-induced lung injury and fibrosis through the down-regulation of nitric oxide metabolites, and proinflammatory and angiogenic cytokines.

## Background

Acute lung injury (ALI) is characterized by diffuse alveolar injury, profound inflammation, increased vascular permeability, and alveolar flooding, which together may result in acute respiratory failure [1,2]. A significant proportion of patients with ALI exhibit severe fibroproliferation 10-14 days after clinical presentation. Presently, no effective therapy for reversing or retarding the fibrotic course of the disease is available, despite the high rates of mortality associated with ALI and fibrosis. One possible way to reduce the rate of mortality is to alter the inflammatory and repair processes that are activated following lung injury. During the past few years, several studies have demonstrated that bone marrow-derived mesenchymal stem cells (BMDMSCs) can localize to and/or participate in the development of new lung tissue [3-5]. In addition, BMDMSC transfer has been attempted as a therapeutic strategy in experimental lung injury and fibrosis. Recent studies involving the administration of stem cells for the treatment of experimental ALI, fibrosis, and emphysema have shown promising results [6,7].

Endotracheal challenge in mice with bleomycin (BLM) represents a well established model of ALI resulting in pulmonary fibrosis that resembles idiopathic pulmonary fibrosis [8,9]. The process occurs in three stages: alveolar epithelial cell death, inflammation, and enhanced collagen deposition with fibroblast and smooth muscle cell proliferation [10-12]. At present, the mechanism by which BLM-induced lung injury is attenuated following mesenchymal stem cell (MSC) administration is unclear. MSCs, which are thought to function as stem cells in the lungs, may limit the injurious effects of BLM by replenishing alveolar epithelial type II cells [4-6], which play various roles in alveolar fluid balance, coagulation/fibrinolysis, and host defense [13].

One alternative mechanism is that MSCs may change the microenvironment of the lung at sites of engraftment [6,14,15], possibly by modulating the production of soluble factors, including transforming growth factor-beta (TGF-β), interleukin (IL)-1α, basic fibroblast growth factor, platelet-derived growth factor, and IL-6, all of which are considered to be possible mediators of lung injury and pulmonary fibrosis [16-20]. The levels of several cytokines, including vascular endothelial growth factor (VEGF), hepatocyte growth factor, and granulocyte colony-stimulating factor, have been shown to increase in the lungs during ALI [21-23]. These cytokines induce bone marrow stem cell mobilization and differentiation both *in vivo *and *in vitro *[14,22,24,25]. In addition to soluble mediators, BLM induces an increase in the production of free radicals, including nitric oxide (NO) [26-28]. NO reacts rapidly with oxygen-centered superoxide radicals to form peroxynitrite, a potent oxidizer that may contribute to tissue injury in ALI and fibrosis [29,30]. We previously reported that the expression of NO synthase increased in a BLM-induced lung injury model [31]. However, few studies have demonstrated a time-dependent effect of stem cells on BLM-induced NO production and inflammatory cytokine expression. Thus, in this study, we measured the levels of various inflammatory cytokines and NO metabolites in BLM-treated rats over time. We also evaluated the therapeutic effects of BDMSC transfer on BLM-induced ALI and fibrosis, and the changes in inflammatory cytokine and NO production.

## Methods

### BM-derived MSCs isolation and in vitro culture-expansion and evaluation of surface marker and potentials to mesenchymal differentiation

**Figure 1:**
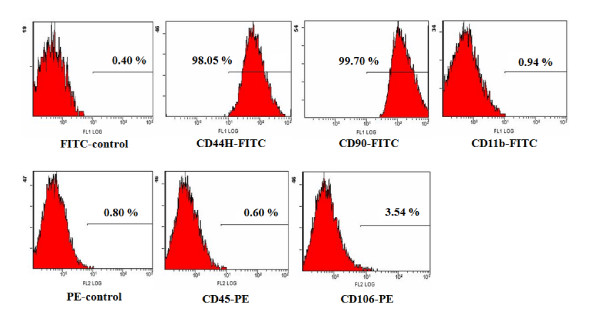
**Flowcytometric Analysis of rat mesenchymal stem cells wased in the study**. The cell surface marker phenotype of these MSCs was shown to be for CD45-, CD106-, CD44H+, CD90+, and CD11b+.

**Figure 2:**
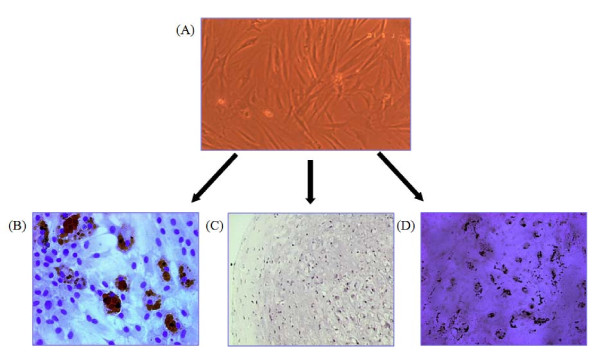
**Variable differential potential of rat mesenchymal stem cells depending on culture method**. (A) Culture-expanded mesenchymal stem cells showed a fibroblast-like morphology following culture expansion *in vitro *(×100). (B) Presence of adipocytes was demonstrated by oil red O staining of cytoplasmic inclusions of neutral lipids (×200). (C) Chondrocytes were demonstrated by toluidine blue staining (×100). (D) Calcium in the differentiated osteocytes was showed by Von Kossa staining (×100).

Fresh bone marrow cells were harvested from the femurs of 6 weeks-old male Sprague-Dawley rats (Charles River Technology Inc.) by flushing DMEM (Gibco BRL, Grand Island, NY, USA) containing 1% penicillin-streptomycin (Gibco BRL) according to the method previously described [32,33]. After washing once with DMEM, the cells were seeded into 75 cm^2 ^tissue culture flask at 1 × 10^6 ^cells/mL in DMEM medium containing 10% fetal bovine serum (FCS, Gibco BRL) supplemented with HEPES (ATCC, Manassas, VA, USA), nonessential amino acids (Cellgro, Herndon, VA, USA), and pyruvate (Invitrogen, Carlsbad, CA, USA), and then cultured at 37°C humidified 5% CO_2 _incubator. After 48 hours, nonadherent cells were removed. The culture medium was changed every 2 days until adherent cells reached subconfluence, then they were detached with 0.25% trypin-EDTA solution, and re-seeded at 4 × 10^3 ^cells/cm^2^. The adherent cells after seventh passage were used for mesenchymal stem cells. The surface phenotypes were examed using antibodies against FITC-conjugated CD44H (Pgp-1, BD Biosciences Pharmingen, San Diego, CA USA), CD90 (Thy-1, BD, Franklin Lakes, NJ, USA), CD11b (Integrin, BD), PE-conjugated CD106 (VCAM-1, BD), and CD45 (BD). The cell surface marker phenotype of these MSCs was shown to be for CD45-, CD106-, CD44H+, CD90+, and CD11b+ (Figure 1). Culture of bone marrow MNCs produced a mono-morphic confluent adherent layer of elongated fibroblast-like cells that survived multiple passages in mesenchymal culture conditions (Figure 2A). Potentials of the rat MSCs were evaluated as differentiation into adipocytes (Figure 2B), chondrocytes (Figure 2C), and osteocytes (Figure 2D). Rat MSCs were induced to differentiate into osteocytes by treating confluent monolayer in DMEM medium containing 0.1 M dexametasone (Sigma), 50 M Ascorbate (Sigma), 10 mM β-Glycerol phosphate (Sigma), 10% FBS for 3 weeks. Osteogenic differentiation was demonstrated by the increase in alkaline phosphatase and the accumulation of calcium. Alkaline phosphatase was detected histologically, and calcium was stained by Von Kossa staining. For chondrogenic differentiation, 2 × 10^5 ^cells were added to 500 ul of DMEM medium containing 10^-7 ^M dexametasone (Sigma), 50 M Ascorbic Acid 2-Phosphate (Sigma), 1 ug/mL transforming growth factor (TGF)-β (R&D System, Mineapolis, MN, USA). The cells were grown as a pelleted micro-mass for 3 wks. The cell pellets were stained with toluidine blue. To induce adipogenic differentiation, rat MSCs were cultured in DMEM medium containing 0.1 M dexamethasone (Sigma), 0.5 mM methyl-isobuthylxanthine (Sigma), 10 g/ml insulin (Sigma), 100 mM indomethacin (Sigma), and 10% FBS (Adipogenic induction medium) for 48-72 hours, And the medium was changed to DMEM medium containing insulin (10 g/ml), and 10% FBS (Adipogenic maintenance medium) for 24 hours. The cells were then re-treated with Adipogenic induction medium. Thereafter the culture was maintained in Adipogenic maintenance medium for 1 week before fixation. Adipogenic differentiation was demonstrated by the accumulation of lipid vesicles by Oil red O (Sigma) staining. At the end of the second passage, bone marrow derived MSCs were successfully differentiated along osteogenic, chondrogenic, and adipogenic lineages, using methods described above (Figure 2).

### Induction of lung injury with BLM and treatment with BMDMSCs

**Figure 3:**
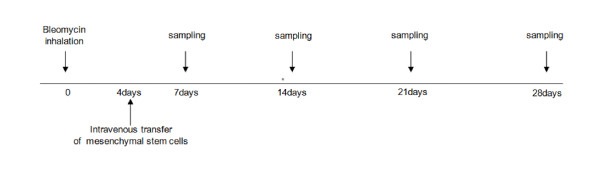
**Schematic diagram of the experimental protocol**. Specific pathogen-free, 6-week-old female SD rats (200-250 g body weight) were treated with 3 mg/kg bleomycin (BLM) dissolved in 5 ml of endotoxin-free water via inhalation. Bone marrow-derived mesenchymal stem cells (1 × 10^6^) were administered via a tail vein on day 4 after BLM treatment. The rats were killed on days 0, 7, 14, 21, and 28. Lung specimens were obtained before and after bronchoalveolar lavage as described in the Materials and Methods.

Specific pathogen-free, 6-week-old female Sprague-Dawley rats (Orientbio Inc., Seongnam-si, Korea) were given 3 mg/kg BLM hydrochloride (Nippon Kayaku, Tokyo, Japan) in 5 ml of normal saline using an ultrasonic nebulizer (NEU12; mean mass median diameter: 4.5 mm, output: 0.15-0.3 ml/min; Omron, Tokyo, Japan; Figure 3) as described previously [31]. On day 4, the rats were anesthetized with isofluorane gas, and 0.1 ml of the BMDMSC suspension (10^7^/ml) was infused via a tail vein. The sham control rats were treated with endotoxin-free water. The animals were maintained at 22°C and 20-50% humidity, with a 12-h light period; food and water were provided *ad libitum*. The animals were housed in a pathogen-free laminar flow cabinet. On days 0, 7, 14, 21, and 28, rats were killed using an overdose of a ketamine (Yuhan Corp., Seoul, Korea) and xylazine (Bayer Corp., Shawnee Mission, KS) mixture. The institutional animal care and use committee of Soonchunhyang University Bucheon Hosptial approved this study.

### Bronchoalveolar lavage (BAL) and preparation of the lung tissues for analysis of the dry/wet ratio, and histological examination

After the animals were killed, the left main bronchus was tied with a string. Following BAL of the right lung, right lungs were removed for protein, or histological analysis. The left lung lobes were removed for wet/dry ratio analysis as previously described [31]. After the wet weight of the excised left lobe was measured, the lobe was placed with a desiccant in an oven at 60°C and reweighed 4 days later. BAL was performed four times using a 1 ml infusion of phosphate-buffered saline (PBS) with withdrawal via a cannula inserted into the trachea. The cells in the bronchoalveolar lavage (BAL) fluid were counted using a hemacytometer. The BAL fluid was centrifuged at 500 × *g *for 10 min and the supernatant was stored at -70°C. Differential cell counts were performed using slides prepared by cytocentrifugation and Diff-quick staining (Scientific Products, Gibbstowne, NJ). Approximately 500 cells were counted. The right lung was removed from each animal and fixed in 4% paraformaldehyde. The specimens were then dehydrated and embedded in paraffin. For histological examination, 4-μm-thick sections were cut using a rotary microtome, placed on glass slides, deparaffinized, and sequentially stained with hematoxylin and eosin (H&E).

### Measurement of collagen using Masson's Trichrome stain and Sircol collagen assay

Sections of lung tissue (4 μm thick) were fixed in Bouin's solution, stained for 1 h at 56°C, washed in tap water for 5 min at room temperature, and stained for 10 min with Weigert's iron-hematoxylin. Masson's thrichrome stain was done as previously described [34]. The total amount of soluble collagen was assessed using a Sircol Collagen Assay Kit according to the manufacturer's instructions (Biocolor, Carrickfergus, Northern Ireland, UK). Briefly, 100 μl of each lung tissue lysate and reference collagen standard were mixed with 1 ml of Sircol dye for 30 min and then centrifuged at 10,000 rpm for 5 min to precipitate the collagen-dye complex. After decanting the suspension, the droplets were dissolved in 1 ml of Sircol alkali reagent and vortexed. The absorbance of the solution was then read at 540 nm. All measurements were performed in quadruplicate. The amount of collagen was calculated from the reference collagen standards, and the minimum detection limit for total soluble collagen was 5 μg/ml. The inter- and intra-assay coefficients of variance were less than 15%. The amount of collagen was expressed as a ratio by normalization to the protein concentration of each specimen.

### Measurement of the IL-1β, TGF-β, VEGF, nitrite, and nitrate concentrations in the lung tissue lysates and of IL-6 and TNF-α in the BAL fluids

The extracted lung tissues were homogenized in a protein lysis solution containing 50 mM Tris-HCl (pH 7.4), 1% NP-40, 50 mM NaCl, 0.5 mM ethylene diamine tetraacetic acid (EDTA), and 100 mM phenylmethylsulfonyl fluoride (PMSF) in distilled water and incubated on ice for 20 min. The protein concentration in each sample was determined using a BCA Kit (Pierce Biotechnology, Rockford, IL). The IL-1β (Immuno-Biological Laboratories Co. LTD, Gunma, Japan), TGF-β (R&D systems, Minneapolis, MN) and vascular endothelial growth factor (VEGF) levels in the lung tissue lysates and IL-6 and TNF-α concentrations in the BAL fluid samples were measured using quantitative sandwich ELISA kits according to the manufacturers' protocols (R&D Systems, Minneapolis, MN, for VEGF; BD Biosciences for IL-6 and TNF-α). The levels of nitrite and nitrate in the BAL fluids were also quantified by ELISA (Biosource International, Camarillo, CA). The minimum detection limits for IL-1β, TGF-β, VEGF, IL-6, TNF-α, nitrite, and nitrate were 1.67 pg/ml, 1000 pg/ml, 1000 pg/ml, 19.5 pg/ml, 15.6 pg/ml, 1.56 μmol/l, and 0.54 μmol/l, respectively. Values below these limits were assigned a value of zero for the purpose of statistical analysis. The inter- and intra-assay coefficients of variance were less than 15%.

### Fluorescence in situ hybridization (FISH)

The localization of male Y chromosome sequences in 2-μm-thick paraffin-embedded sections of rat lung tissue was performed using an ID Labs Kit and a mouse Y chromosome paint probe (London, England, UK) as described previously [33,35]. The sections were counterstained with 4', 6-diamidino-2-phenylindole and 1, 4-phenylenediamine in PBS and glycerol (125 ng/ml) and photographed under a Leica (Deerfield, IL) RX-DMV upright fluorescent microscope attached to a digital camera (Cooke Sensicam, Melville, NY).

### Statistical analysis

The data are expressed as the mean ± standard error. SPSS version 10.0 (SPSS, Chicago, IL) was used to perform all statistical analyses. The study groups were compared using the Kruskal-Wallis test. When significant differences were found, the Mann-Whitney U-test was used to compare the two samples. Differences were considered significant at *p *< 0.05.

## Results

### Effect of BMDMSC transfer on BLM-induced pulmonary edema, body weight and mortality

**Figure 4:**
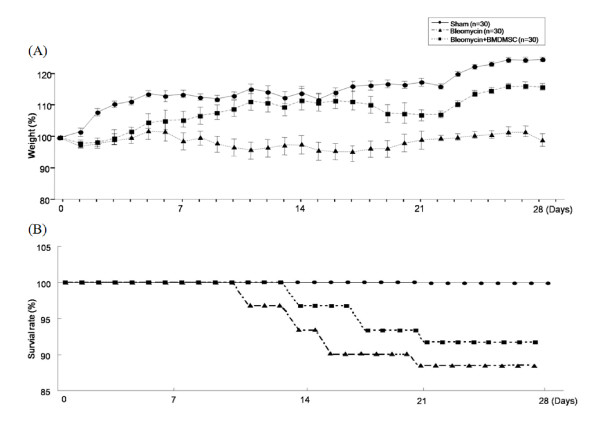
**Effect of BMDMSC transfer on body weight and survival rate**. (A) BMDMSC transfer increased body weight gain in the bleomycin - treated rats. (B) Bleomycin - treated rats showed higher mortality rate compared to the sham - treated rats while BMDMSC transfer attenuated the mortality of the bleomycin - treated rats.

**Figure 5:**
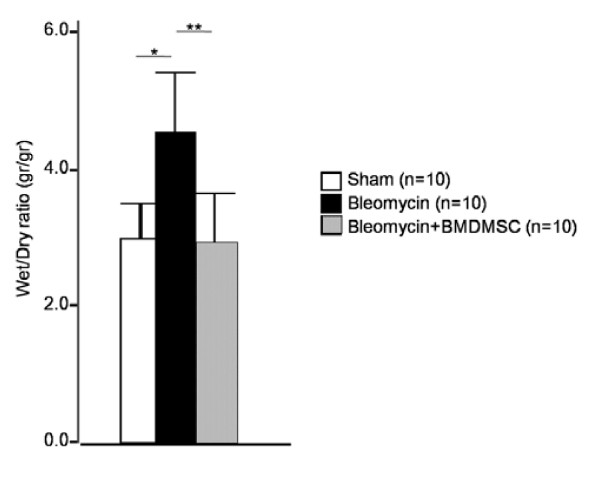
**Effect of bone marrow-derived mesenchymal stem cell (BMDMSC) transfer on the bleomycin (BLM)-induced increase in the lung wet/dry ratio**. The transfer of BMDMSCs significantly reduced the BLM-induced increase in the wet/dry ratio on day 7. * and ** indicate *p *< 0.05 and *p *< 0.01, respectively.

Sham rats continuously gained weight till the end of the study (28 day) while bleomycin - treated rats did not gain weight. BMDMSC transfer increased weight in the bleomycin - treated rats (Figure 4A). We decided to begin the investigational phase of the study 1 week after the start of intravenous infusion of BMDMSC (Figure 4B). In the bleomycin - treated rats (n = 30), the rats died consistently every week. In contrast, although some rats in the BMDMSC transferred rats died in the first 10 days after. Bleomycin-treated rats showed higher mortality rate compared to the sham - treated rats while BMDMSC transfer attenuated the mortality of the bleomycin - treated rats (*p *< 0.05; Figure 4B). To quantitatively assess the degree of pulmonary edema following BLM treatment, the wet/dry weight ratio of the left lung was measured in each animal. The BLM-treated rats had a significantly higher wet/dry weight ratio compared to the sham-treated rats at day 7 (4.5 ± 0.4 vs. 3.6 ± 0.5, *p *= 0.04; Figure 5); however, BMDMSC transfer significantly decreased the ratio in the BLM-treated rats to the level in the sham-treated rats (2.9 ± 0.3, *p *= 0.009).

### Effect of BMDMSC transfer on the BAL cell profile and lung histology in the BLM-treated rats

**Figure 6:**
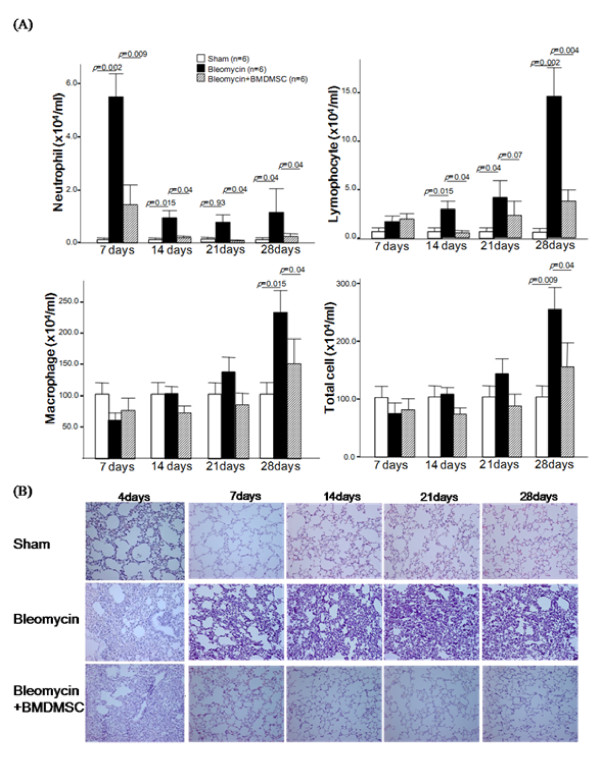
**(A) Time-dependent changes in the inflammatory cell profile in bronchoalveolar lavage (BAL) fluid**. Bleomycin (BLM)-induced increases in the number of neutrophils, lymphocytes, and macrophages in the BAL fluid were significantly reduced between days 7 and 28 by the transfer of BMDMSCs. * and ** indicate *p *< 0.05 and *p *< 0.01, respectively. (B) Effect of BMDMSC transfer on BLM-induced histologic changes in the lungs. Lung tissues were stained with hematoxylin & eosin on days 7, 14, 21, and 28. BLM treatment induced the intra-alveolar and interstitial infiltration of inflammatory cells. The extent of inflammation significantly decreased from day 7 to day 28 following BMDMSC transfer. Magnification = × 200.

The number of neutrophils in the BAL fluid of the BLM-treated rats peaked at day 7 (*p *= 0.002) then steadily was higher from day 14 to day 28 compared to the number in the sham rats (*p *< 0.05; Figure 3A); however, BMDMSC transfer significantly restored the BLM-induced increase in the number of neutrophils to nearly the level in the sham-treated rats. The number of lymphocytes rose significantly at day 14 (*p *= 0.015) and continued to increase up to day 28 following BLM treatment (*p *= 0.002). A similar significant increase in the number of macrophages was observed at day 28 in the BLM-treated rats compared to the sham-treated rats (*p *= 0.015; Figure 6A). The transfer of BMDMSCs significantly reduced the BLM induced-increases in the number of lymphocytes and macrophages in the BAL fluid (*p *< 0.05; Figure 6A), but the suppressive effect was incomplete.

Histological analysis using H&E staining revealed exudative changes and heavy infiltration of polymorphonuclear and mononuclear inflammatory cells such as neutrophils and lymphocytes into the intra-alveolar and interstitial spaces following BLM treatment from day 7 to day 28 (Figure 6B). The transfer of BMDMSCs markedly reduced the infiltration of inflammatory cells to the extent observed in the sham-treated rats (Figure 6A).

### Effect of BMDMSC transfer on collagen deposition in the lung tissues and the total amount of soluble collagen in lung extracts

**Figure 7:**
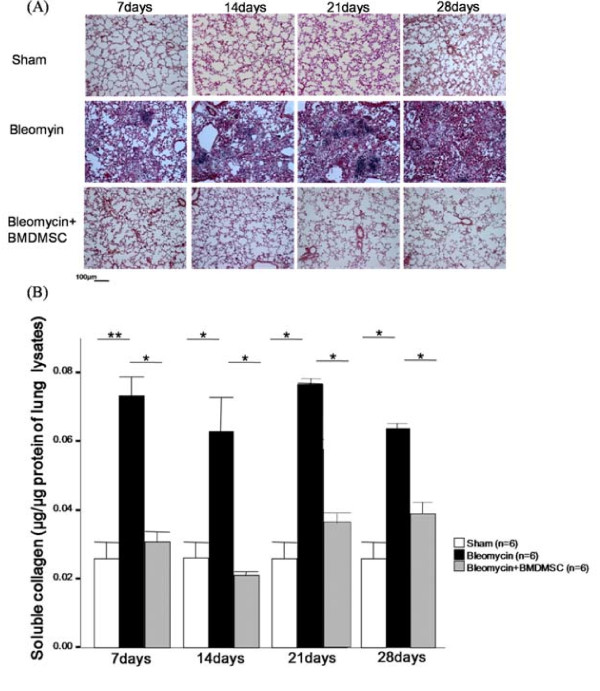
**Effect of BMDMSC transfer on collagen deposition in lung tissues and the total amount of soluble collagen in lung extracts**. (A) Collagen deposition in the lungs was analyzed using Masson's Trichrome stain. (B) The Sircol collagen assay was used to measure the total amount of soluble collagen in lung tissue lysates. * and ** indicate *p *< 0.05 and *p *< 0.01, respectively.

To analyze collagen deposition in the lung, Masson's Trichrome stain and the Sircol collagen assay were applied to lung tissue sections and lysates, respectively. Collagen deposition was detected in the interstitium of the lungs following BLM treatment from day 7 to day 28. The transfer of BMDMSCs nearly abrogated the deposition of collagen throughout the entire experimental period (Figure 7A). Our histologic findings were confirmed by an analysis of the total amount of collagen in lung tissue lysates by ELISA. The total amount of collagen was doubled on day 7 following BLM treatment, and was then maintained at the same level up to day 28 (*p *= 0.071-0.004; Figure 7B). The transfer of BMDMSCs reduced the BLM-induced increase in the amount of collagen to the level detected in the sham-treated rats (*p *= 0.04-0.016; Figure 7B).

### Effect of BMDMSC on IL-6 and TNF-α in BAL fluid samples and IL-1β, TGF-β VEGF and NO metabolites in lung extracts

**Figure 8:**
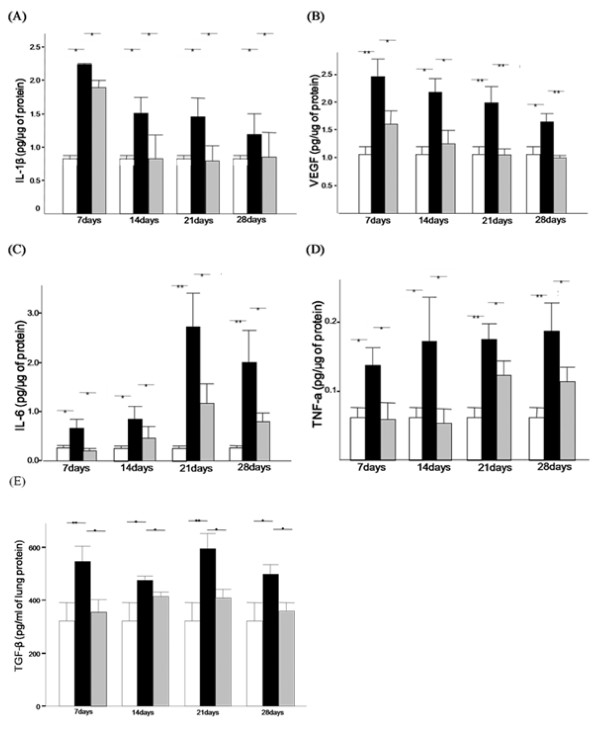
**Effect of bone marrow-derived mesenchymal stem cell (BMDMSC) transfer on the levels of IL-6 and TNF-α in bronchoalveolar lavage fluid and IL-1β, vascular endothelial growth factor (VEGF), and TGF-β in lung lysates**. Bleomycin treatment significantly increased the levels of IL-1β (A), VEGF (B), IL-6 (C), TNF-α (D), and TGF-β (E) between days 7 and 28. The increases in IL-1β, VEGF, IL-6, TNF-α and TGF-β were significantly reduced by the transfer of BMDMSCs. * and ** indicate *p *< 0.05 and *p *< 0.01, respectively.

The level of IL-1β and VEGF in lung extracts prepared from the BLM-treated rats peaked at day 7, and then decreased slightly up to day 28. The level of TGF-β in the lung extracts of the BLM-treated rats was maintained throughout time course. All values were significantly elevated compared to those in the sham-treated rats (*p *= 0.019-0.029, *p *= 0.015-0.002). The transfer of BMDMSCs completely suppressed the increase in IL-1β, TGF-β and VEGF after BLM treatment to the level in the sham rats (*p *= 0.029-0.04, *p *= 0.052-0.004; Figure 8). The level of IL-6 in the BAL fluid of the BLM-treated rats was also two times higher than that in the sham-treated rats at day 7 (0.67 ± 0.18 vs. 0.26 ± 0.05 pg/μg of protein, *p *= 0.04). The level then rose steadily up to day 21 (2.73 ± 0.69 pg/μg of protein, *p *= 0.002) and remained relatively constant up to day 28 (2.03 ± 0.36 pg/μg of protein, *p *= 0.002). The transfer of BMDMSCs significantly reduced the BLM-induced increase in IL-6 during the entire experimental period (*p *= 0.04-0.026), but the suppressive effect was incomplete (Figure 8C).

A similar increase in TNF-α following BML treatment was observed. The level of TNF-α in the BAL fluid of the BLM-treated rats rose significantly from day 7 to day 28, becoming twice of that in the sham-treated rats (*p *= 0.04-0.003; Figure 8D). BMDMSC transfer also restored the BLM-induced increase in TNF-α to the level in the sham rats at days 7 and 14, but had only a partial effect at days 21 and 28.

**Figure 9:**
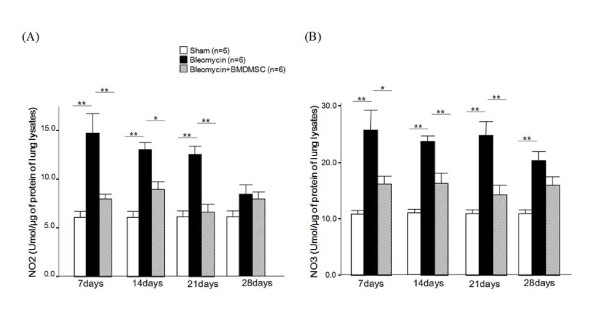
**The effect of bone marrow-derived mesenchymal stem cell (BMDMSC) transfer on the bleomycin (BLM)-induced increases in nitrate and nitrite in lung extracts**. BLM (n = 6) increased the nitrate (A) and nitrite levels (B) in lung extracts from day 7 to day 21; however, the effect was significantly reduced by BMDMSC transfer (n = 6). * and ** indicate *p *< 0.05 and *p *< 0.01, respectively.

The presence of NO metabolites, including nitrite and nitrate, indicates *in vivo *NO production in the airways and lungs [36]. Increased nitrite and nitrate concentrations were detected in lung extracts from the BLM-treated rats compared to the levels in the sham-treated rats (*p *= 0.002) at day 7 (Figure 9); thereafter, the levels tended to decrease. The transfer of BMDMSCs significantly reduced the BLM-induced increase in nitrate and nitrite from day 7 to day 21 (*p *= 0.026-0.004).

### Engraftment of donor-derived cells in the lungs after BMDMSC transfer

**Figure 10:**
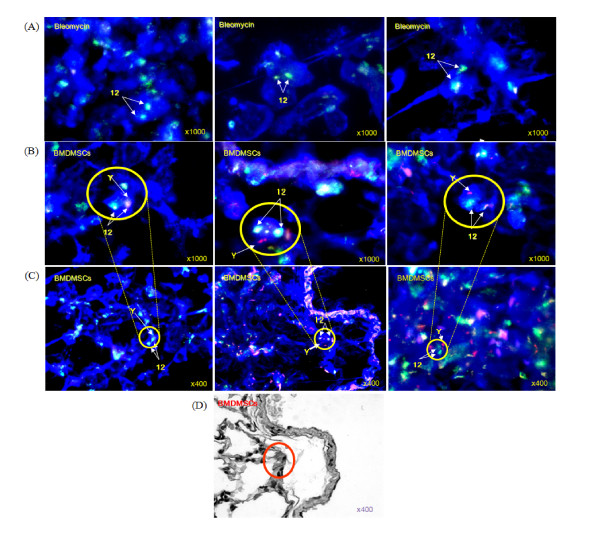
**Localization of the Y chromosome in bleomycin (BLM)-treated rats following bone marrow-derived mesenchymal stem cell transfer**. A 2-μm-thick section of lung tissue from a BLM-treated rat hybridized with a fluorescein isothiocyanate-conjugated Y chromosome paint probe and counterstained with ethidium bromide is shown. (A) Bleomycin treated rats (n = 3). (B) Bone marrow-derived mesenchymal stem cell in recipient lungs under fluorescent microscope two weeks after BMDMSC injection in BMDMSC transfer rats (n = 3). The arrows indicate nuclei containing a Y chromosome. Magnification = 1000×. (C) Donor cells containing the Y chromosome zoomed out pictures of the lung. Magnification = 400×. (D) Localization of the cells containing the Y chromosome in serial section of lung tissue of BMDMSC transfer rats. H&E stain, magnification = 400×.

To demonstrate the localization of the infused BMDMSCs to the lungs, FISH using a rat Y chromosome paint probe was used to identify the engraftment sites of the male donor cells in the lungs of the BMDMSC-transferred rats. Relatively few cells were detected in lung tissue that demonstrated a positive hybridization signal against the Y chromosome. Donor cell showing Y chromosome mainly localized in the alveolar epithelium of the bleomycin (BLM)-treated rats following bone marrow-derived mesenchymal stem cell transfer (Figure 10B and 10C). Donor-derived cells were sparsely detected at day 28 in the lung tissues of the BLM-treated rats. On the representative H&E stained serial section of lung tissue, donor cell showing Y chromosome mainly localized in the alveolar epithelium of the bleomycin (BLM)-treated rats following bone marrow-derived mesenchymal stem cell transfer (Figure 10D).

## Discussion

The disease course of ALI is marked by three phases, exudative, proliferative, and fibrotic, although inflammatory and repair mechanisms occur in parallel, rather than in series [1,2]. The exudative phase encompasses the first 7 days after injury, while the proliferative phase spans days 7-21, and the fibrotic phase occurs 2-4 weeks after the initial pulmonary injury [1,2]. Previous experiments in mice have demonstrated the protective effect of BMDMSC transfer against BLM-induced fibrosis in the lungs of animals 14 days after BLM treatment when given at the beginning of ALI [6,15,23]. This prompted us to evaluate the protective effect of this therapeutic strategy against fibrosis, as well as against exudation and inflammation in a time-dependent manner. In the present study, BLM treatment increased the level of vascular permeability as demonstrated by the increased wet/dry ratio at day 7 (Figure 3). Thereafter (days 14-28), the increase was unremarkable (data not shown). This and our previous data [31] indicate that BLM maximally increases vascular permeability at an early stage of lung injury. On the same day (day 7), lung inflammation was neutrophil-dominant as reflected by BAL cell analysis and lung histological analysis. The neutrophilic inflammation later changed to lymphocyte- and macrophage-dominant inflammation (days 21-28). Note that the amount of collagen, which was assessed quantitatively and qualitatively, peaked at day 7 then remained constant up to day 28. This indicates that inflammation and collagen deposition started concomitantly at a very early stage of ALI following BLM administration. Fibroproliferation is also an early response to lung injury in human acute respiratory distress syndrome (ARDS) and is an important clinical marker for late-stage survival [37]. Although the pattern of lung inflammation changed over time, the degree of fibrosis reflected the increased level of collagen deposition throughout the study period. In the present study, BMDMSC transfer at day 4 following BLM inhalation significantly reduced the BLM-induced increase in the wet/dry ratio, neutrophilic infiltration, and collagen deposition to nearly the levels in the sham-treated rats. These data indicate that BMDMSC transfer may be effective and curative against the ongoing inflammation-induced alveolar damage caused by BLM inhalation.

In the present study, BMDMSCs from male Sprague-Dawley rats were transferred to female recipients 4 days after the inhalation of BLM. In our previous study, peak levels of pulmonary edema and neutrophilic inflammation were detected between days 4 and 14 after BLM inhalation [31]. This suggests that the condition of the lungs in the BLM-treated rats at day 4 resembles clinical ARDS in humans. Thus, we decided to transfer the BMDMSCs 4 days after BLM treatment. Ortiz et al. [6] observed that the administration of MSCs immediately after challenge with BLM reduced the extent of inflammation within the lung, but was ineffective when transferred 7 days after BLM challenge. We currently have no explanation for the ineffectiveness of BMDMSC treatment in late stage of lung injury.

BLM-induced lung injury is characterized by capillary leakage and alveolar edema, which are hallmarks of ALI [15-20,25]. The overexpression of VEGF in murine lung induces widespread intra-alveolar edema, suggesting that increased pulmonary vascular permeability in the early stages of ALI may be caused, at least in part, by VEGF overexpression [38]. VEGF was up-regulated in a murine lipopolysaccharide (LPS)-induced ALI model, and the changes in the balance between VEGF, angiopoietin-1, and angiopoietin-4 after LPS exposure may modulate the influx of neutrophils, protein leakage, and alveolar flooding in ALI mice [39]. In addition to VEGF, the levels of TNF-α and IL-6 were also elevated in the BLM-treated rats. TNF-α and IL-6 have multiple effects on acute inflammation and infiltration by neutrophils and lymphocytes [1,2]. TNF-α also contributes to the pathophysiology of interstitial lung disease by inducing the apoptosis of epithelial cells and the sequential release of TGF-β, IL-1β, and IL-1 receptor antagonist (IL-1ra) [40]. In addition, the production of reactive oxygen and nitrogen species is related to apoptosis in alveolar epithelial cells [41], the release of TGF-β from pulmonary epithelial cells [42], and the activation of TGF-β1 through the disruption of its interaction with latency-associated peptide [43]. In patients with idiopathic pulmonary fibrosis (IPF), the expression of iNOS is elevated in the lungs [44]. In our previous study of a BLM lung injury animal model, iNOS was differentially expressed [31]. These findings prompted us to assess whether changes in IL-1β, VEGF, NO metabolite, and pro-inflammatory cytokine including IL-6 and TNF-α production occur in BLM-treated rats following BMDMSC transfer to evaluate the mechanism of the protective effect of BDMSC transfer against ALI/fibrosis. IL-1β and VEGF was elevated in the lung lysates following BLM treatment in the present study. Note that the levels of IL-1β, VEGF, and nitric oxidative stress peaked at day 7 and then decreased steadily. This result is in agreement with the observed peak change in the wet/dry ratio of the lung. Thus, IL-1β, VEGF, and nitric oxidative stress may be associated with early changes in vascular permeability in ALI. As seen by the change in the wet/dry ratio in the BLM-treated rats, BMDMSC transfer almost fully suppressed the BLM-induced increase in these mediators throughout the experimental period (Figure 8 and 9). These data indicate that BMDMSC transfer may be effective against increases in vascular permeability via the regulation of IL-1β, VEGF, and NO stress. In addition to the mediators measured in our study, BMDMSCs may exert their therapeutic effects against various types of lung injury through other cytokines and mediators. Rojas et al. [23] demonstrated the protective effect of BMDMSC transfer against the increase in circulating levels of G-CSF and GM-CSF with a decrease in inflammatory cytokines, including IL-2, INF-γ, and IL-4, following BLM-induced lung injury in mice. G-CSF and GM-CSF are well known for their ability to promote the mobilization of endogenous stem cells. The administration of MSCs directly into the airspace down-regulated the pro-inflammatory response to endotoxin by reducing the levels of TNF-α and MIP-2 in both the BAL fluid and plasma while increasing the level of the anti-inflammatory cytokine IL-10 [45]. In addition, the beneficial effect of MSCs is independent of the ability of the cells to engraft in the lung as observed in our study, and is unrelated to the clearance of endotoxin by MSCs [45].

BLM induces lung epithelial cell death, followed by acute neutrophilic influx, chronic inflammation, and parenchymal fibrosis within 4 weeks in susceptible strains of mice [46]. Thus, we recorded our observations up to day 28 to evaluate the later stages of ALI/fibrosis. In contrast to the complete effect of the BMDMSCs on neutrophilic inflammation, lymphocyte and macrophage infiltration progressively increased over time in our experiments, even in the BMDMSC recipients. At the same time, the effect of BMDMSC against the BLM-induced increase in IL-6 and TNF-α was incomplete. Lymphocytosis in the lung lesions of patients with idiopathic interstitial pneumonia is associated with an increased amount of IL-6 in the lung [47]. This indicates that the transfer of BMDMSCs may exert a partial effect on chronic inflammation at a late stage. We don't know the meanings of the partial response at the moment. Before infusing the BMDMSCs, we checked their purity using CD44H and CD45. Only those cells that were over 95% positive for CD44H were used in our experiments. Thus, contamination with other cell types was avoided. BMDMSCs are pluripotent CD45^-^, CD44H^+ ^adherent cells that are capable of differentiating into a variety of cell types, including endothelial, epithelial, and neuronal cells, as well as adipocytes, depending on the culture conditions [33,48]. In the lung, BMDMSCs can differentiate into type I and type II alveolar epithelial cells, endothelial cells, fibroblasts, and bronchial epithelial cells [4-6]. Our cytogenetic data revealed the presence of donor cells in the alveolar walls in the later phase of BLM-induced ALI, but very sparsely (Figure 10). Thus, our data suggest that MSCs may alter the microenvironment of the lung at the engraftment sites as demonstrated by the other study [45]. However, when considered that the donor-derived cells were apparently sparsely were detected from day 7 to 21 and the overall engraftment level were very low through the entire experimental period, the anti inflammatory and anti fibrotic effects of the MSCs might be a result of the systemic anti-inflammatory effects of MSCs.

## Conclusions

The systemic administration of BMDMSCs at the early stage effectively abolished the neutrophilic lung inflammation and collagen deposition that is typically observed following BLM treatment in animal models. Transfer of BMDMSCs down-regulates the BLM-induced increase in levels of IL-1β, TGF-β VEGF, IL-6, TNF-α, and NOS in the lung through the late stage of lung injury. These data suggest that BMDMSC transfer may be an effective strategy for the treatment of lung injury and fibrosis via modulation of microenvironment of injured lung.

## Competing interests

The authors declare that they have no competing interests.

## Authors' contributions

SHL performed all experimental steps and wrote the manuscript; ASJ provided experimental assistance and wrote the first draft of the manuscript; YEK carried out the immunoassays of the NO_2 _and NO_3_; JYC, THK and SJ helped to submit the manuscript. SKP and JHW isolated the BM-derived MSCs and evaluated of mesenchymal differentiation; YKL carried out the FISH; YHK and CSP conceptualized of the study and supervised this project. All authors read and approved the final manuscript.
